# Gene transfer: anything goes in plant mitochondria

**DOI:** 10.1186/1741-7007-8-147

**Published:** 2010-12-22

**Authors:** John M Archibald, Thomas A Richards

**Affiliations:** 1 Canadian Institute for Advanced Research, Program in Integrated Microbial Biodiversity, Department of Biochemistry and Molecular Biology, Sir Charles Tupper Medical Building, Dalhousie University, Halifax, NS B3H 1X5, Canada; 2 Department of Zoology, Natural History Museum London, Cromwell Road, London, SW7 5BD, UK

## Abstract

Parasitic plants and their hosts have proven remarkably adept at exchanging fragments of mitochondrial DNA. Two recent studies provide important mechanistic insights into the pattern, process and consequences of horizontal gene transfer, demonstrating that genes can be transferred in large chunks and that gene conversion between foreign and native genes leads to intragenic mosaicism. A model involving duplicative horizontal gene transfer and differential gene conversion is proposed as a hitherto unrecognized source of genetic diversity.

See research article: http://www.biomedcentral.com/1741-7007/8/150

## Horizontal gene transfer in plants

**Figure 1:**
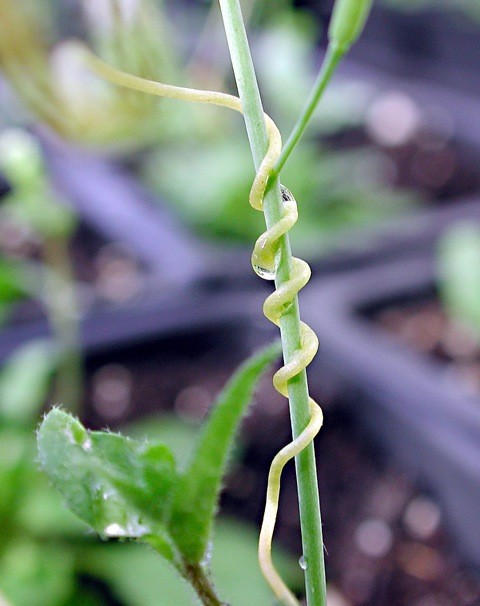
**The parasitic plant *Cuscuta *wrapping around one of its many possible hosts, *Arabidopsis***. Image kindly provided by Dr Collin Purrington, Swarthmore College.

Among the most profound biological insights gleaned from the past decade of comparative genomics has been the realization that horizontal gene transfer (HGT) has impacted the genetic make up of virtually everything we have chosen to sequence. Long recognized as a major force in the evolution of prokaryotic genomes [1] and somewhat more recently for microbial eukaryotes [2], HGT can also impact the genomes of complex, multicellular organisms: case in point, plant mitochondrial DNA (mtDNA). Parasitic plants and their hosts (Figure 1) have proven to be avid donors and recipients of mtDNA [3], and two recent studies, one in the pages of *BMC Biology *[4], have provided new mechanistic detail on the causes and consequences of plant HGT [4,5]. HGTs involving plant nuclear and chloroplast DNA appear (for now at least) to be rare [6,7]. In contrast, plant mtDNA appears to be highly mobile, a fact that has significant practical and theoretical implications for plant biology.

The first suggestions that plant mitochondria might be exceptional in terms of DNA uptake came from studies of their fungal-derived, homing group I introns (for example, [8]), and a growing body of evidence for plant-to-plant HGT has since accumulated [3]. The current champion is *Amborella trichopoda*, a 'primitive' flowering plant found exclusively on the island of New Caledonia, the mtDNA of which is littered with foreign genes acquired from both angiosperm and non-angiosperm donors [9,10].

How exactly does plant HGT happen? An important clue comes from the fact that while examples of ancient and 'recent' HGT events involving chloroplast DNA have been documented [2], such events appear to be very infrequent [6]. This makes sense given that plant mitochondria possess active DNA uptake systems and are capable of fusion; chloroplasts do not and are not (see [3] and references therein). Thus, given direct physical contact between host and parasite tissue, ample opportunity for mtDNA uptake and exchange would seem to exist. Yet despite extensive phylogenetic evidence supporting the notion that plant mitochondrial HGT is rampant, numerous mechanistic uncertainties remain. These include the question of whether DNA or RNA is the donor molecule and whether a virus or some other vector mediates the transfer.

## Gene transfer, gene conversion, and intragenic mosaicism

Mower *et al. *[4] sought to elucidate the process of plant-to-plant HGT by using PCR to survey mitochondrial genes in species of the parasitic plant *Cuscuta *(Figure 1) and one of its many hosts, the dicot weed *Plantago*. After initially casting a wide net to capture ten protein and RNA genes from both *Cuscuta gronovii *and *Plantago coronopus *mtDNA, they sequenced three genes, *atp1*, *atp6 *and *matR*, from a range of host and parasite relatives and showed that each gene appears to have been transferred recently (within the last few million years) from the mitochondrial genome of *Cuscuta *to that of *Plantago*. In and of itself this is no longer surprising, but the authors demonstrate that (i) the three genes appear to have been transferred together in the context of a relatively large fragment of DNA (and not RNA, which can be inferred due to the presence of unedited cytidine residues at sites known to undergo RNA editing); (ii) both native and 'foreign' homologs (xenologs), the latter in the form of pseudogenes, co-exist in several species; and (iii) multiple gene conversion events have occurred between co-resident loci.

Hao *et al. *[5] have gone even further. They uncovered a 'gorgeous mosaic' of multiple mitochondrial genes and gene fragments in various plant host-parasite lineages, including a striking chloroplast-to-mitochondrion transfer involving a region of *atp1*. In total, approximately one-third of the HGT events investigated (5 of 17 genes) appear to have involved at least some gene conversion - the non-reciprocal exchange of DNA between homologous sequences - suggesting that this process plays an important role in the integration of foreign genetic material. The true significance of HGT-associated gene conversion may in fact be underestimated because of differential gene loss, lack of phylogenetic resolution and insufficiently sensitive detection methods. The authors propose a model - duplicative HGT and differential gene conversion (DH-DC) - in which intra- and inter-organellar gene transfer and recombination are creative forces in the generation of mitochondrial genetic diversity.

By duplicative HGT, Hao *et al. *mean the transfer and integration of a foreign set of genes, a single gene, or a gene fragment from donor to recipient mtDNA that does not instantly replace its endogenous counterpart. Herein lies the key to the model, as the co-existence, however transient, of foreign and native loci within the same subcellular compartment allows gene conversion to occur. Gene conversion is a well-understood process (for example, as a generator of allelic diversity during meiosis), and in the context of DH-DC, gene conversion, occurring in either a continuous or discontinuous manner, gives rise to patchwork recombinants. Such heterogeneity is not phenotypically 'silent': the recombinant *atp1 *and *matR *genes uncovered by Hao *et al. *yield proteins with different amino acid sequences [5].

## DH-DC in plant mtDNA: impact and implications

The results of Mower *et al. *[4] and Hao *et al. *[5] are both troubling and satisfying. Troubling because recombination between resident and foreign gene copies, no matter how transient the latter, has the potential to 'wreak havoc' on the results of even the most cautious phylogeneticist intent on inferring organismal phylogenies from plant mitochondrial genes [5]. In addition, mutation rate estimates for plant mtDNA - painstakingly obtained and long considered to be exceptionally low [3,11] - should, in the face of the DH-DC model, now be considered overestimates: a certain fraction of single-nucleotide differences observed between sequences will be due to gene conversion, not point mutation. Ultimately, though, satisfaction comes from a deeper understanding and appreciation of the true complexity of plant mtDNA evolution.

More generally, Hao *et al. *[5] raise the intriguing possibility that DH-DC could be a driver of gene evolution in prokaryotes, one that has thus far gone undetected. And what of nuclear genomes? In concluding their study of *Plantago*-*Cuscuta *HGT, Mower *et al. *[4] state that '...unravelling this history will probably require sequencing multiple mitochondrial and nuclear genomes from *Plantago*.' Given the pace at which sequencing technologies are evolving, it is hard to imagine this not happening in the near future, not just within plants but also for microbes and multicellular organisms across the full breadth of eukaryotic diversity. With the exception of specific lineages such as fungi, whose nuclear genomes are being sequenced at both shallow and deep evolutionary divergences, the field of comparative genomics is still in 'gap filling' mode. The Hao *et al. *[5] and Mower *et al. *[4] studies underscore the fact that when it comes to HGT the devil is in the details: only in the context of meticulous comparisons of both closely and distantly related genomes is a deep understanding of the pattern, process and full scope of eukaryotic HGT likely to emerge.
